# Heritability of Attractiveness to Mosquitoes

**DOI:** 10.1371/journal.pone.0122716

**Published:** 2015-04-22

**Authors:** G. Mandela Fernández-Grandon, Salvador A. Gezan, John A. L. Armour, John A. Pickett, James G. Logan

**Affiliations:** 1 Department of Disease Control, London School of Hygiene and Tropical Medicine, Keppel Street, WC1E 7HT, London, United Kingdom; 2 School of Forest Resources and Conservation, University of Florida, Gainesville, Florida, 32611, United States of America; 3 School of Life Sciences, University of Nottingham, Nottingham, NG7 2UH, United Kingdom; 4 Biological Chemistry and Crop Protection Department, Rothamsted Research, Harpenden, AL5 2JQ, United Kingdom; New Mexico State University, UNITED STATES

## Abstract

Female mosquitoes display preferences for certain individuals over others, which is determined by differences in volatile chemicals produced by the human body and detected by mosquitoes. Body odour can be controlled genetically but the existence of a genetic basis for differential attraction to insects has never been formally demonstrated. This study investigated heritability of attractiveness to mosquitoes by evaluating the response of *Aedes aegypti* (=*Stegomyia aegypti*) mosquitoes to odours from the hands of identical and non-identical twins in a dual-choice assay. Volatiles from individuals in an identical twin pair showed a high correlation in attractiveness to mosquitoes, while non-identical twin pairs showed a significantly lower correlation. Overall, there was a strong narrow-sense heritability of 0.62 (SE 0.124) for relative attraction and 0.67 (0.354) for flight activity based on the average of ten measurements. The results demonstrate an underlying genetic component detectable by mosquitoes through olfaction. Understanding the genetic basis for attractiveness could create a more informed approach to repellent development.

## Abstract 2

A twin study finds that there is a correlation in our attractiveness to mosquitoes by odour and our genetics, providing evidence that the likelihood of getting bitten is heritable.

## Introduction

Attractiveness to biting insects is important in medical contexts, mostly in the dynamics of transmission of pathogens by mosquitoes that cause diseases such as dengue and malaria. Blood feeding is an essential part of the lifecycle for most mosquito species as it provides females with the proteins necessary for egg production.

It has been reported that, when selecting a human host, mosquitoes have a preference for certain individuals [1]. Various factors contribute to differential attractiveness to biting insects. For example, it has been shown that pregnant woman are significantly more attractive to *Anopheles gambiae* (the principal malaria vector in Africa) than their non-pregnant counterparts [2]. Similarly, in what may be a case of host-parasite manipulation, it is seen that people infected with malaria become more attractive to *A*. *gambiae* during the transmissible stage of infection [3]. Individuals with a greater body mass do appear to be more attractive to mosquitoes and midges [4,5], which may relate to other characteristics such as increased surface area and CO_2_ output. A similar case is likely to be seen with higher body temperatures which, along with increased relative humidity, improves the convection and effective distribution of the odours produced [6,7] and are not necessarily attractive to mosquitoes themselves.

The most frequently offered anecdotal explanation for differential attraction is diet. Amongst these are claims that consumption of garlic, vitamin B or beer will repel mosquitoes. A double-blinded trial has demonstrated that garlic was ineffective as a repellent against *Aedes aegypti* [8], with a similar result seen for vitamin B when tested with *Anopheles stephensi* [9]. The consumption of beer has been shown to increase biting incidence of *A*. *gambiae* over alcohol-free control groups [10]. Work on the biting midge has also failed to find diet as a factor [5]. So far there has been no clear and consistent dietary explanation for the differential attraction which is seen throughout these studies. From an evolutionary perspective, it may be that the selection pressure on the mosquito to detect odour changes which would be associated with the host in a highly variable and transient fashion are not sufficiently strong and precedence would be given to cues which more consistently represent a suitable host.

Although recent work has demonstrated that differential attractiveness to mosquitoes is due to differences in body odour [1,11,12] the mechanisms underpinning this have not yet been studied. It is known that some chemicals used during host location are produced by skin bacteria [13], but it is also possible that compounds are produced directly via skin cells through biosynthetic pathways. In either case, human odour is known to be controlled, at least in part, by genetic factors [14], and it is possible that variation in our attractiveness to mosquitoes is also modulated via the same mechanism(s).

Investigating the genetic control of attractiveness to biting insects could lead to the development of novel insect repellents, and individualised strategies for avoiding insect bites could be formulated rationally if the genetic basis for variation between individuals were known. Understanding genetically-determined mechanisms that underlie variation in the production of repellents by individuals could also lead to the development of novel methods to enhance the production of natural repellents by human beings, thus creating a novel repellent technology that could minimise the need for topical application.

Currently, little is known about the genetic basis of variation in human attractiveness to biting insects. A questionnaire study on adolescent twins demonstrated a greater reporting of frequent bites among relatives of more susceptible individuals, and greater concordance for monozygotic (MZ) than for dizygotic (DZ) twin pairs, suggesting a genetic influence [10]. Similarly Logan *et al*., [5] demonstrated a significant interaction between the frequencies of bites a first child receives and how often the parent is usually bitten, which suggests the mechanism responsible for making a person attractive or unattractive to mosquitoes is heritable. It is likely that the production of human volatiles that attract or repel mosquitoes shows genetic variation; however, the above studies relied on self-reported values and never have been experimentally tested in controlled conditions.

We aimed to identify if there is an underlying genetic component to our attractiveness to biting insects. Through a series of behavioural assays using identical and non-identical twins we studied correlation of genetic relatedness and level of attractiveness to *Aedes aegypti*.

## Results

**Fig 1:**
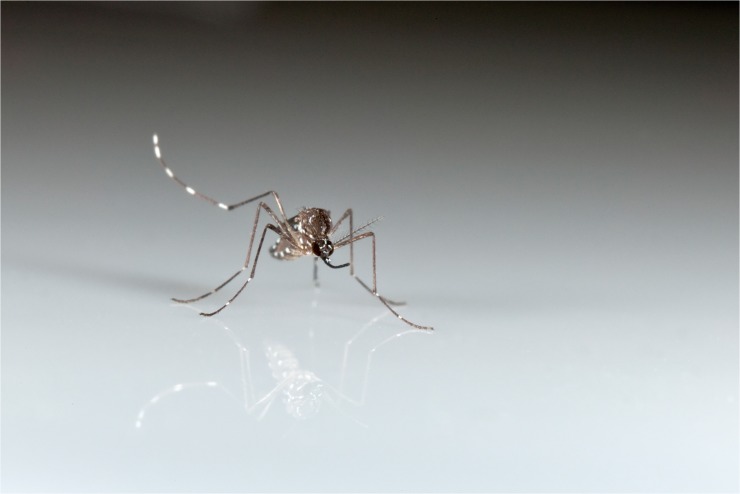
Female *Aedes aegypti* mosquito. Female *A*. *aegypti* mosquitoes used in the experiment to test attractiveness to odours from the hands of identical and non-identical twins.

**Fig 2:**
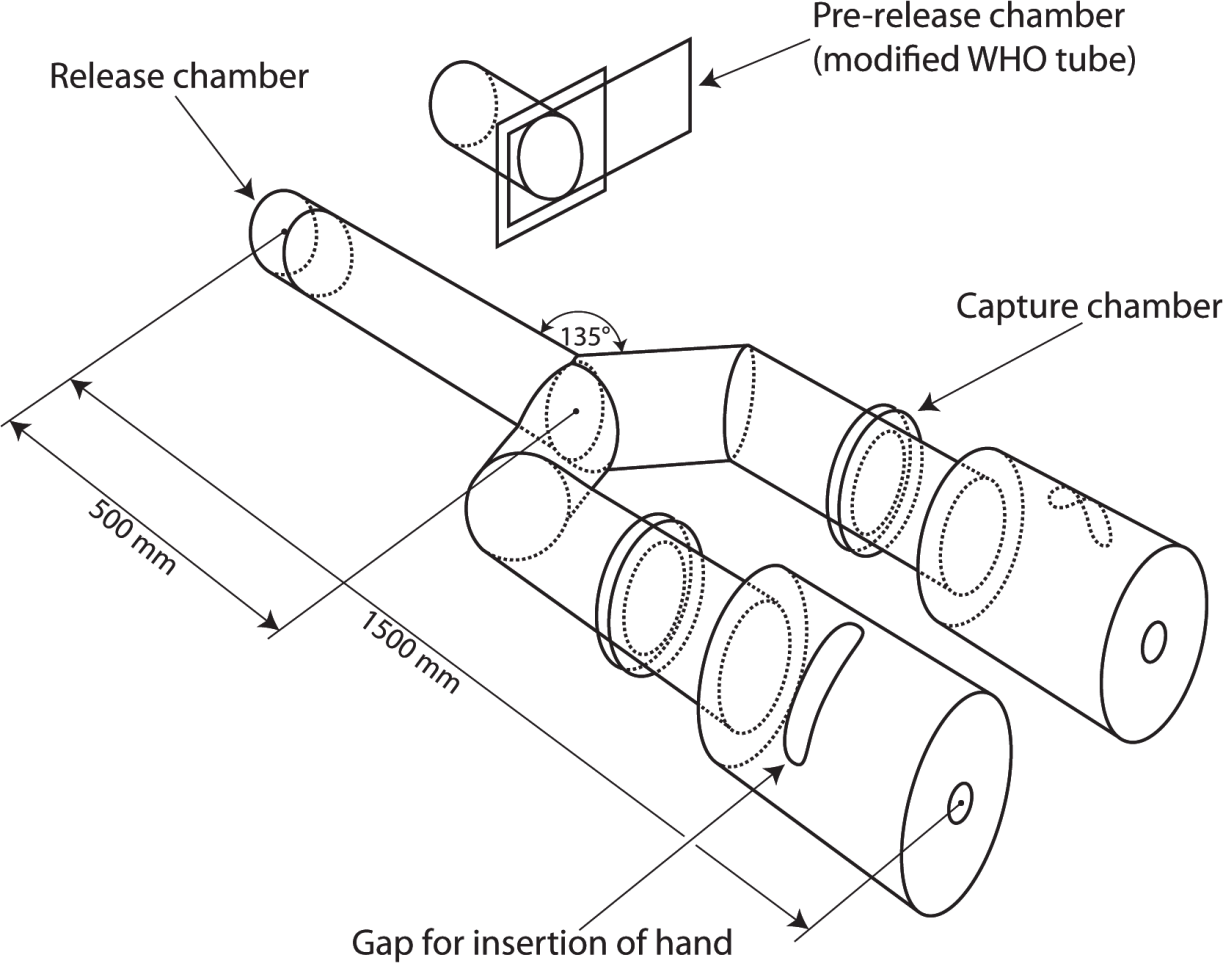
The Y-tube olfactometer. Olfactometry equipment used to test the behavioural response of female *Aedes aegypti* mosquitoes to odours from the hands of identical and non-identical twins. The Y-tube formed of plexiglass with a 70 mm internal diameter allowing the free flight of mosquitoes within the airflow.

We tested the hypothesis that attractiveness to mosquitoes has heritable factors by investigating the response of *Aedes aegypti* (= *Stegomyia aegypti*) mosquitoes (the vectors of dengue; Fig 1) to volatile odours from the hands of sets of monozygotic and dizygotic twins using a laboratory-based Y-shaped olfactometer test (“the Y-tube”, Fig 2).

The Y-tube olfactometer allows investigation of behavioral responses of mosquitoes to stimuli by blowing air through the Y-shaped tube and allowing mosquitoes to fly upwind and make a choice between a stimulus in one arm of the Y-tube and another stimulus (or control) in the other arm in a controlled laboratory setting.

Groups of 20 female *A*. *aegypti* mosquitoes (Fig 1) were flown in a Y-tube olfactometer. In each trial a different choice of odour could be presented to the mosquitoes; the treatment combinations were as follows: 1) twin A vs clean air; 2) twin B vs clean air; 3) twin A vs twin B; and 4) clean air vs clean air (negative control). A total of 18 identical (monzygotic) and 19 non-identical (dizygotic) female twin pairs were tested with each pair completing 10 replicates of each treatment set. For each replicate a new batch of mosquitoes was used. The response variables analysed corresponded to: 1) upwind flight activity (the proportion of mosquitoes that flew upwind beyond 30 cm in the stem of the Y-tube); and 2) relative attraction (the proportion of mosquitoes in the arm of the Y-tube that contained the odour stimulus).

**Fig 3:**
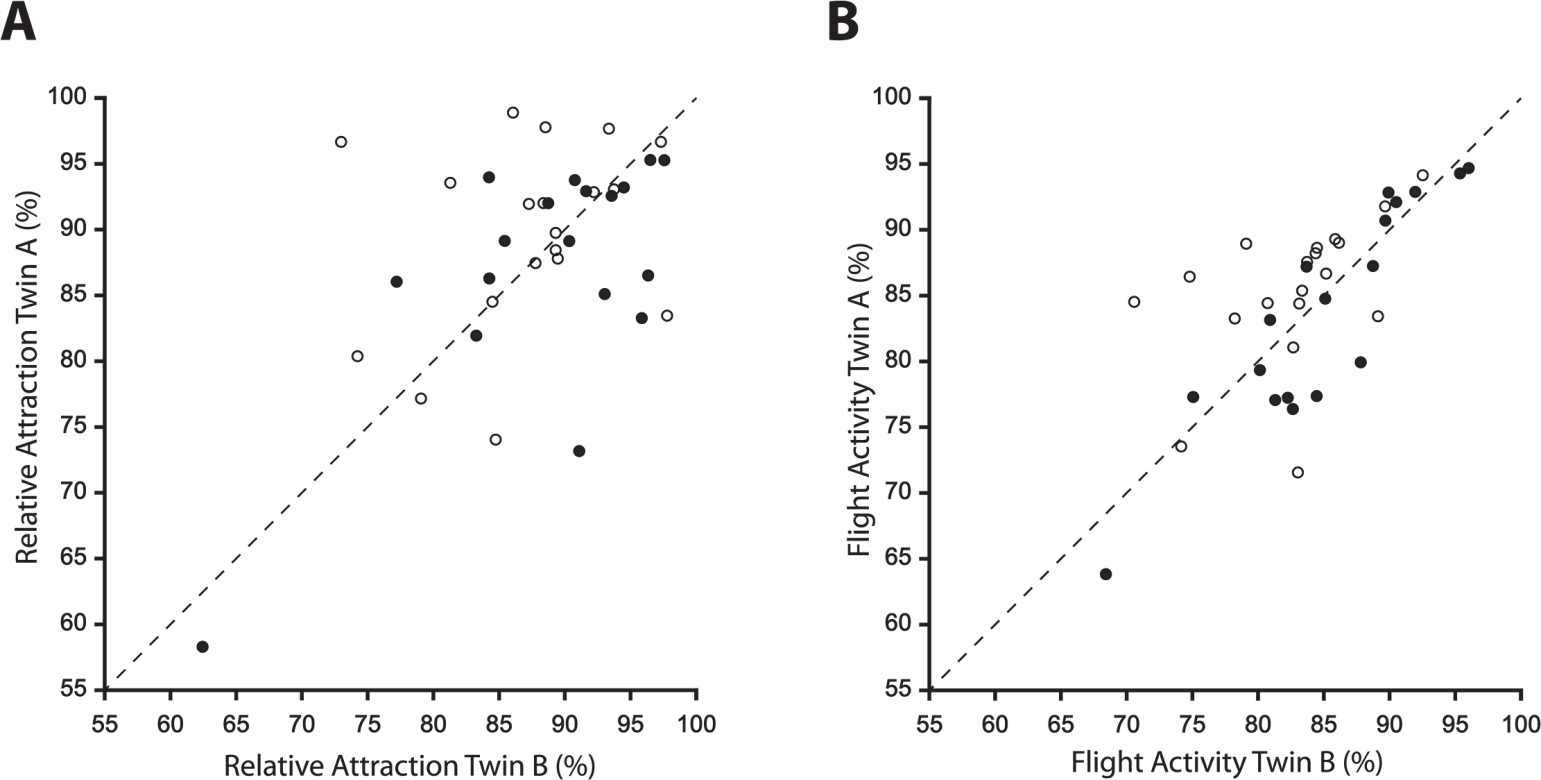
Scatter plots for (A) relative attraction and (B) flight activity for dizygotic twins (open circles) and monozygotic twins (closed circles).

Bootstrap correlation analysis demonstrated, for the trait relative attraction, a stronger correlation between monozygotic twins (r = 0.563) than for dizygotic twins (r = 0.287) (Table 1 and Fig 3).

**Table 1:** Intraclass correlations (r) and narrow-sense heritability (h^2^).

	Mosquito behaviour	
	Relative attraction	Flight activity
**r (95% CI)**		
**Dizygotic twins**	0.29 (-0.29–0.07)	0.48 (0.07–0.83)
**Monozygotic twins**	0.56 (-0.08–0.92)	0.90 (0.76–0.97)
**h** ^**2**^ **(SE)**		
**Twins tested separately**	0.62 (0.124)	0.67 (0.354)
**Twins tested together**	0.83 (0.096)	-

Shown for the behaviours, relative attraction and flight activity, which were used to determine attractiveness to mosquitoes. The estimated h^2^ values for “Twins tested separately” were obtained using data where a twin was evaluated individually against a control. The h^2^ values for “Twins tested together” were estimated based on raw averages from trials where both twins were paired in the same run.

The negative control showed no bias in mosquito preference when treatment was not present with a mean relative attraction of 0.486 and 0.508 on MZ and DZ days respectively. Furthermore, flight activity showed significant positive correlations with a larger value for monozygotic twins (r = 0.896) than dizygotic twins (r = 0.479) (Table 1 and Fig 3). Additionally, the absolute differences in relative attraction between twin pairs resulted in a larger variance for DZ of 42.01 in comparison to MZ with a value of 23.79; however, these variances were not significantly different (P = 0.25). In contrast, for the absolute difference in flight activity, a much larger variance was found for DZ against MZ with values of 15.91 against 5.39 respectively, which were significantly different (P = 0.03). Because both the DZ and MZ estimates of correlation and heritability were subject to sampling error, their confidence limits are relatively wide, especially with a small number of twin pairs, as in this study.

The estimated values for narrow-sense heritability were 0.62 and 0.67 for relative attraction and flight activity, respectively when the twins were tested separately (Table 1). In contrast, the heritability, when the twins were tested together, resulted in a higher value of 0.83 for relative attraction (Table 1).

Exploration of the individual data (i.e. single measurements) indicated that associations with treatment arm (left or right), or body temperature, were not significant (p-value > 0.05) for either of the responses.

## Discussion

Our results demonstrate an underlying genetic component to the human odour profile, a genetic difference that is detectable by mosquitoes through our odour and used during host selection.

The relatively small total sample size, and the nature of the response variable analyzed, places limits on the precision of our conclusions. Hence, all of these heritability values would put attractiveness to biting insects at a level similar to traits such as human height (h^2^ ≈ 0.8) and IQ (h^2^ ≈ 0.5–0.8) [15]. Although the power of genome-wide studies depends on the genetic architecture of a given trait, our estimate of h^2^ is high enough to warrant further genomic studies to investigate and identify the specific genes involved in differential attractiveness of human beings to mosquitoes.

It is not known whether the differences between MZ and DZ twins is due to the presence or absence of attractive or repellent chemicals, as demonstrated by Logan *et al*.[11], but the chemistry associated with the genes involved should be subject to further investigation. Until the mechanism is elucidated we do not know whether differential attractiveness has evolved from selective pressures or as a by-product of metabolic processes [16]. However, it is plausible that the mechanism exists as a natural defence strategy against biting insects and ultimately the pathogens that they transmit. Odours are known to be controlled, at least in part, by genetic factors. Indeed the body odours of twins have been shown to be similar [14]. Odour cues associated with genetic similarity are thought to be controlled via the major histocompatibility complex (MHC) genes—often cited as being potentially involved in mate selection and inbreeding avoidance [17; 18; 19]. MHC-related odours are believed to be produced from MHC-derived peptides or their metabolites, microflora on the skin, or via metabolism of peptides by the microflora [20; 21]. With a strong cross-over of MHC-related odours and the source of odours that elicit behavioural responses in mosquitoes, this is a potential target for further genetic investigations.

The variation in chemical production could also have influences from non-heritable components such as gut or skin commensal flora. A recent study analyzed skin emanations and blood samples of volunteers, alongside MHC profiling, to investigate genes associated with attractiveness but could not identify a significant association [22].

Physical and molecular information about the twins used in this study, including genome-wide genotyping, is available for several of the participants. Further work is now necessary to identify the genetically-controlled mechanisms underlying the variable production of volatile compounds that make individual humans more or less attractive to biting insects.

## Materials and Methods

### Mosquitoes

All mosquitoes used in the trials were *A*. *aegypti* females aged between 5–7 days old. Prior to the experiment mosquitoes were maintained at 27±2°C, 70% humidity and allowed to feed only on glucose solution. Mosquitoes were stored in 30 x 30 cm fabric cages (Bugdorm, Taiwan) before being collected in WHO chambers (Product: WHO/VBC/81.806) and placed in the laboratory environment to acclimate. Mosquitoes were collected in groups of approximately 20 using a mouth aspirator one hour before commencing the experiments.

### Volunteers

Ethical approval for this study was granted by the London School of Hygiene and Tropical Medicine Observational / Interventions Research Ethics Committee (approval number A421). A total of 18 identical and 19 non-identical female twin pairs, between the age of 50 and 90, were selected from the TwinsUK database from the Department of Twin Research to participate in the study. Only post-menopausal women were selected for the study to eliminate the variation introduced by sex or phase of menstruation. All participants were given a full explanation of the test procedure both verbally and in written form. Written consent was provided by all participants with the guidelines for consent approved by the Observational / Interventions Research Ethics Committee. For 24 hours prior to the experiment the volunteers were asked to avoid alcohol and strong smelling foods such as garlic, onions and chili. Participants were randomly assigned as twin A or twin B. Participants were asked to remove all jewelry from the hands and were supervised as they washed their hands thoroughly with simple odour-free soap (Unilever, UK) and a nailbrush. After washing their hands, volunteers were instructed not to touch anything and allow their hands to air dry. Once participants’ hands were dry the assay commenced.

### Bioassay

A plexiglass Y-tube olfactometer was used to assay mosquito odour preferences. Air was pushed through a glass bell jar (5 l) containing distilled water (1 l) and a charcoal filter before being split through two flowmeters and adjusted to ensure each arm of the Y-tube received 10 L per minute of filtered air to produce a mean speed of 0.13 m sec^−1^ in the arms and 0.11 m sec^−1^ in the stem of the olfactometer.

To commence the assay, a WHO chamber containing approximately 20 female *A*. *aegypti* mosquitoes was attached to the release chamber and connected to the stem of the olfactometer. The slider on the WHO chamber was opened while the release chamber gate was closed. At this point mosquitoes were able to enter the Y-tube apparatus and receive the odours available upwind but were unable to fly towards the source. After 30 s in this position the gate was opened and 90 s was given for mosquitoes to complete an upwind flight into one of the arms of the olfactometer. At the end of the trial the number of mosquitoes in each area of the olfactometer was recorded. Two behaviours were recorded: 1) upwind flight activity (the proportion of mosquitoes that flew upwind beyond 30 cm in the stem of the Y-tube); and 2) relative attraction (the proportion of mosquitoes in the arm of the Y-tube that contained the odour stimulus). After each trial the apparatus was disassembled and the mosquitoes removed using a Medicair vacuum cleaner (Miele International, Germany). Ten replicates were completed in this way for each treatment set. Temperature and relative humidity were maintained at 27 ± 2°C °C and 60%, respectively.

The response to the hand of each participant was compared with a negative control of clean air simultaneously in the other side of the Y-tube. Both twins, within a pair, were also tested against each other by inserting the hand of one twin in one side of the Y-tube and the other twin in the other side. The order of testing was randomised and each twin was randomly allocated to a specific side of the Y-tube between replicates to avoid positional bias. In summary, the treatment combinations evaluated were: 1) twin A vs clean air; 2) twin B vs clean air; 3) twin A vs twin B; and 4) clean air vs clean air (negative control). During control runs the “inlet chamber” was wrapped in PTFE tape (Fisher Scientific, UK). This tape was removed to allow the participants to insert their hands into the Y-tube. The individuals from each twin pair were tested on the same day and the order of testing each twin or twin/control combination was randomised using a Latin Square design. Only one set of twins was tested per day.

Since mosquitoes are known to respond differentially to heat sources and it is possible that differences in skin temperature may have caused the differential responses seen here; we investigated this by measuring skin temperature of each volunteer using a surface thermometer (Braun, Germany) before each behavioural test.

### Statistical Analyses

Two subsets of the dataset were identified for the statistical analyses. The first dataset included the data from each of the twins tested individually against a control of clean air (hereafter called independent data). This dataset provides an independent comparison of the response of each individual. The other dataset consisted of data where twins were paired against each other in the trials (hereafter called paired data). Hence, this dataset provides a comparison between twins.

The independent data were analysed using traditional animal breeding statistical tools following Visscher *et al*. [23]. Here, a relationship matrix that describes the level of additive relatedness between individuals (0.5 and 1 for DZ and MZ, respectively) was incorporated into the linear mixed model as a pedigree file. This model used the average of 10 measurements for each twin and considered a random effect of individual (additive effects) and twin pair (common environment). This model fitted to each of the response variables provided estimated variance components that were used to calculate narrow-sense heritability. All models were fitted using restricted maximum likelihood (REML) as implemented in the package ASReml v. 3 [24]. Standard errors of these heritability estimates were obtained using the delta method.

For the above data, Bootstrap correlation coefficients, based on 100 draws, for each type of twin (DZ and MZ) were obtained to evaluate the significance of the correlation estimates for the responses average relative attraction and flight activity. This method was chosen as it provides robust estimates. Also, sample variance estimates were compared for DZ and MZ twins for the absolute difference between twin pairs for relative attraction and flight activity and the variables for the DZ and MZ using an F-test based on a 5% significance level. This test had the null hypothesis that both of the variances for DZ and MZ were identical. A larger variance for DZ than MZ twins is an indication that the former pairs are more diverse, probably due to genetic factors.

For the paired data, the average of 10 measurements for the response relative attraction was considered. Narrow-sense heritability [25] was estimated using the following expressions:

Var(A)=Var(DZ)-Var(MZ)

$$Var\left(T\right)=Var\left(DZ\right)-\frac{Var\left(MZ\right)}{2}$$

$$h^{2}=\frac{Var(A)}{Var(T)}$$

where A, T, DZ and MZ correspond to the additive, total, dizygotic and monozygotic terms. Note that the above expression uses the estimated variance of the DZ and MZ twins separately. The resulting heritability assumes that the variance due to common environment is negligible, and therefore, it represents an upper bound for heritability, as the latter is a term that should be added to Var(T). As before, the standard error of this heritability was obtained using the delta method.

## Supporting Information

S1 Data Raw data for both dizygotic and monozygotic twins from Y-tube olfactometer experiment. Twin assignments of A and B were at random. Pair means combines the data for use in analysis with values provided for relative attraction and flight activity.
(XLSX)
